# Evaluation of the First Commercial Hepcidin ELISA for the Differential Diagnosis of Anemia of Chronic Disease and Iron Deficiency Anemia in Hospitalized Geriatric Patients

**DOI:** 10.5402/2012/567491

**Published:** 2012-02-29

**Authors:** Inge Geerts, Pieter Vermeersch, Etienne Joosten

**Affiliations:** ^1^Laboratory Medicine, University Hospitals Leuven, Herestraat 49, 3000 Leuven, Belgium; ^2^Internal Medicine, Division of Geriatric Medicine, University Hospitals Leuven, Herestraat 49, 3000 Leuven, Belgium

## Abstract

*Introduction*. Anemia is a frequent problem in hospitalized geriatric patients, and the anemia of chronic disease (ACD) and iron deficiency anemia (IDA) are the 2 most prevalent causes. The aim of the study was to assess the possible role of serum hepcidin in the differential diagnosis between ACD and IDA. *Methods*. We investigated serum hepcidin, iron status, anemia, and C-reactive protein in 39 consecutive geriatric patients with ACD and IDA. Serum hepcidin levels were determined using a commercial ELISA kit (DRG Instruments, Marburg, Germany). We also measured hepcidin in 26 healthy controls. *Results*. The serum hepcidin levels were not significantly higher in the 28 patients with ACD as compared to the 11 patients with IDA. *Conclusions*. The serum hepcidin levels measured using the commercial ELISA kit (DRG) do not appear to increase in older patients with ACD. It should be noted that an assay-specific problem could explain our results.

## 1. Introduction

The anemia of chronic disease (ACD) and iron deficiency anemia (IDA) are the 2 most prevalent causes of anemia in hospitalized geriatric patients [1]. An inflammatory process plays a central role in the pathogenesis of the ACD with low serum iron and normal or increased iron stores, while the absence of iron stores is the hallmark of the IDA [2]. However, the differential diagnosis between these two causes of anemia is often difficult in clinical practice. The diagnosis is even a greater challenge in case of the combination of ACD and IDA in the same patient. Serum ferritin has been the most commonly used single laboratory parameter to discriminate between ACD and IDA [2]. However, there is no agreement on the lower reference value, and its level increases in response to an underlying inflammatory process. The clinical significance of other laboratory parameters such as the serum transferrin receptor remains unclear and is not widely used [3]. A bone marrow investigation has been considered by some investigators as the gold standard to assess the iron status, but others have found that this investigation is not always diagnostic for iron deficiency [4]. Research on the pathogenesis of ACD has recently focused on the role of hepcidin, a 25 amino acid peptide synthesized in the liver and triggered by inflammatory stimulators such as interleukin-6. This peptide appears to be a key regulator of iron homeostasis [5].

## 2. Patients and Methods

Our study group comprises 39 consecutive elderly patients (mean age 83.4 y, 32 females and 7 males) with ACD and IDA and admitted to the acute geriatric ward of our hospital. Anemia was defined as a hemoglobin concentration <11.5 g/dL for men and women. In order to assess the clinical significance of the hepcidin analysis, we chose to define IDA and ACD according to stringent but noninvasive criteria. Eleven patients were considered to have IDA defined as a serum ferritin level ≤20 *μ*g/L. Twenty-eight patients were considered to have ACD, and they fulfilled all of the following criteria: serum ferritin >100 *μ*g/L, serum transferrin saturation <16%, C-reactive protein (CRP) >10 mg/L, and the presence of an acute or chronic inflammatory process (acute pneumonia 11, urinary tract infection 7, cancer 6, chronic osteomyelitis 1, decubitus ulcer 2, giant cell arthritis 1). Patients with a serum creatinine >2 mg/dL, another established cause for the anemia, or treated with iron or blood transfusion during the last 2 months were also excluded. Peripheral blood counts, CRP, serum transferrin saturation, serum iron, serum ferritin, and serum creatinine were determined in morning specimens collected after an overnight fast according to routine laboratory analyses. For the measurement of hepcidin, a serum aliquot was frozen immediately at −70°C. Serum levels of hepcidin were determined in batch using a commercial ELISA kit (DRG Instruments, GmbH, Marburg, Germany) according to the manufacturer's protocol. The within and between assay coefficient of variation is <4.9% and <11.5%, respectively. The dynamic range of the assay is between 0.9 to 140 ng/mL. The 5 to 95% range in apparently normal healthy adults is 13.3 to 54.4 ng/mL according to the manufacturer. We also studied a group of 26 healthy volunteers (mean age 46 y, 4 men and 22 females). These controls were not anaemic (hemoglobin concentration >13 g/dL for men and >12 g/dL for women), had a ferritin level >16 *μ*g/L (women) and >30 *μ*g/L (men) and a CRP <5 mg/L.

Statistical Analysis: Means were compared by student's *t*-test for the evaluation of the baseline characteristics, and the 1-way analysis of variance was used for the statistical calculations between the various groups (SPSS 19). Log transformed data were used for serum ferritin, transferrin saturation, CRP, and serum hepcidin levels. This study was approved by the ethical committee of the University Hospitals Leuven.

## 3. Results

**Figure 1:**
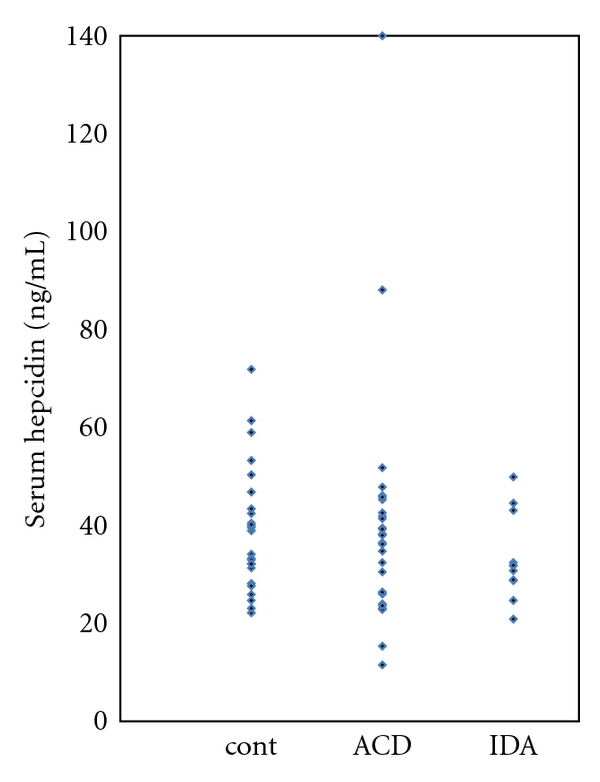
Serum hepcidin levels in 26 healthy controls (cont), 28 patients with anemia of chronic disease (ACD) and 11 patients with iron deficiency anemia (IDA).

The characteristics of the 11 patients with IDA and the 28 patients with ACD are shown in Table 1. It is remarkable that the serum hepcidin levels were comparable between the patients with IDA and ACD. The highest hepcidin level in the IDA group was 49.9 ng/mL, and only 3 out of the 28 patients in the ACD group had a higher level (see Figure 1). The CRP level was <5 mg/L in 8 and >10 mg/L in 3 patients with IDA. When the total study group was arbitrarily subdivided in those with (CRP level >20 mg/L) and without (CRP level ≤20 mg/L) inflammation as we also did in our previous study [3], the geometric mean for the serum hepcidin level in the 25 patients with a CRP level >20 mg/L was 35.2 ng/mL (95% range 17 to 72 ng/mL) and 32.8 ng/mL (95% range 12 to 90 ng/mL) in the 13 patients with a CRP level <20 mg/L (*P* = 0.61).

The geometric mean for the hepcidin concentration in the 26 healthy volunteers was 28.1 ng/mL (5 to 95% range 20.5 ng/mL to 66 ng/mL) which was not statistically different  compared to the hepcidin levels in patients with ACD and IDA (ANOVA, *P* = 0.53).

## 4. Discussion

We found comparable serum hepcidin levels in patients with ACD and IDA. The 5 to 95% reference interval in our control population was comparable to the data delivered by the manufacturer. It is noteworthy that none of our control subjects had clinical or laboratory signs of inflammation or iron deficiency.

Our results are, however, not in accordance with four recent studies [6–9]. Ganz et al. found that the serum hepcidin levels were undetectable in 18 of the 19 patients with iron deficiency (serum ferritin <10 *μ*g/L) but significantly elevated in patients with inflammation defined as a CRP >10 mg/L. There is no information regarding the ferritin concentration in patients with inflammation in this study [6]. In another study, Theurl et al. showed that patients with ACD had significantly higher serum hepcidin levels than patients with IDA, ACD/IDA, and controls [8]. Due to the definition of subpopulations in the study by Theurl et al., ferritin was also significantly different between the subgroups of ACD with and without iron deficiency [8]. Thomas et al. studied a 155 anaemic patients with latent iron deficiency and reported that serum hepcidin can differentiate patients with IDA from ACD and ACD/IDA. Serum hepcidin correlated with ferritin and ferritin index [9]. On the other hand, urinary hepcidin levels were not correlated with proinflammatory markers in a large sample of an older general population [7].

There are several limitations to our study. Our study population is by its nature a heterogeneous group of vulnerable geriatric patients. Aging is associated with increased inflammation and a mild rise in inflammatory markers such as IL-6, but additional laboratory analyses were not performed. A higher CRP level is common in hospitalized older patients with IDA [3], and 3 out of the 11 patients with IDA had a CRP >10 mg/L. We are also aware that the number of patients is limited, but we doubt whether a larger group could significantly alter our results. Although there are no standard criteria for the diagnosis of ACD and IDA, we chose to define our study groups according to well-established laboratory criteria. Finally, several mass spectrometry and immunochemical methods have been developed for hepcidin quantification in serum and urine. Currently, there is no reference method for hepcidin measurements. A generally accepted reference interval is not yet available at this moment, nor a validated cutoff for the diagnosis of IDA or ACD. In the study by Kroot et al., several quantitative methods were compared [10]. The analytical variation of the methods was comparable and was low for all methods [10]. However, the hepcidin concentrations in urine and serum differed considerably between all methods. These differences are suggested to be caused by (i) the use of different calibrators, (ii) possible hepcidin aggregation in the sample or the standard solution, (iii) measuring only free, only bound, or both fractions of hepcidin, or (iv) by interference of hepcidin isoforms [10]. The commercial DRG ELISA assay used in this study was not included in the comparison study of Kroot et al. [10].

In conclusion, this is the first study that investigates the possible role of serum hepcidin for the diagnosis of different types of anemia in a geriatric hospitalized population using a commercially available DRG ELISA kit. According to other recent studies, we would expect higher hepcidin levels in ACD and lower levels in IDA patients as compared to our control group. The reason for this is not clear, although an assay-related problem that could explain these inadequate results is possible. However, at this moment it is premature to draw firm conclusions, and further investigation is needed.

## Figures and Tables

**Table 1:** Characteristics of the patients with iron deficiency anemia and anemia of chronic disease. M: male; F: female; IDA: iron deficiency anemia; ACD: anemia of chronic disease; MCV: mean corpuscular volume; CRP: C-reactive protein.

	IDA	ACD	*P-*value
Number (M/F)	11 (2/9)	28 (5/23)	
Age (years), mean (SD)	81 (7.3)	84 (5.8)	0.2
Hemoglobin (g/dL), mean (SD)	8.9 (2.1)	10.1 (0.8)	0.1
MCV (fL), mean (SD)	80 (6)	91 (3.4)	<0.001
Ferritin (*μ*g/L), geometric mean (95% range)	13.3 (5.6–32)	250 (88–715)	<0.001
Transferrin saturation (%), geometric mean (95% range)	5.9 (2–17)	10.1 (4.6–22)	<0.001
CRP (mg/L), geometric mean (95% range)	4 (0.4–52)	49 (4.6–515)	<0.001
Hepcidin (ng/mL), geometric mean (95% range)	32.4 (19.5–54)	35.3 (13.7–90)	0.58
